# A Case of Dermatomyositis Causing Cryptogenic Organizing Pneumonia

**DOI:** 10.7759/cureus.6296

**Published:** 2019-12-05

**Authors:** Jeffrey A Miskoff, Rana Ali, Moiuz Chaudhri

**Affiliations:** 1 Internal Medicine, Jersey Shore University Medical Center, Neptune City, USA; 2 Internal Medicine, Hackensack Meridian Health Jersey Shore University Medical Center, Neptune City, USA; 3 Internal Medicine, Shore Pulmonary, Ocean, USA

**Keywords:** chronic organizing pneumonia, bronchiolitis obliterans organizing pneumonia, interstitial lung diseases, chronic inflammatory conditions, dermatomyositis

## Abstract

Cryptogenic organizing pneumonia (COP), also known as idiopathic bronchiolitis obliterans organizing pneumonia (BOOP), is a rare inflammatory condition. It often presents as sequelae of existing chronic inflammatory diseases such as rheumatoid arthritis, systemic lupus erythematosus, and various connective tissue conditions. This case describes a 28-year-old African American female who presented with a complex clinical picture involving chronic inflammatory processes and the pulmonary system. The initial evaluation suggested pneumonia to be the underlying cause of respiratory symptoms; however, ultimately, a diagnosis of BOOP with dermatomyositis was made.

## Introduction

Cryptogenic organizing pneumonia (COP) is the idiopathic form of organizing pneumonia, previously known as bronchiolitis obliterans organizing pneumonia (BOOP). It is a form of diffuse interstitial lung disease (ILD) affecting the bronchioles, alveolar ducts, and surrounding lung tissue and structure [1]. A secondary form of organizing pneumonia is associated with connective tissue diseases and chronic inflammatory conditions such as rheumatoid arthritis and dermatomyositis. Terminology “organizing” refers to unresolved pneumonia, which has transformed from alveolar exudates to fibrotic changes within the alveoli [2]. The purpose of this case is to 1) increase awareness of this rare condition and 2) highlight the importance of interdisciplinary communication among the specialists and the primary care clinician to manage this complex disorder.

## Case presentation

A 28-year-old African American female with a past medical history of connective tissue disease, pituitary adenoma, hypothyroidism, uterine fibroids, and ovarian cysts presented with normal respiratory status until April 2015, when she started to experience dyspnea. Her dyspnea worsened to the point where she could not walk across the room without coughing and feeling short of breath. At that time, the patient visited urgent care and started on levofloxacin for respiratory tract infection. Subsequently, she developed arthralgia along with joint swelling.

**Figure 1:**
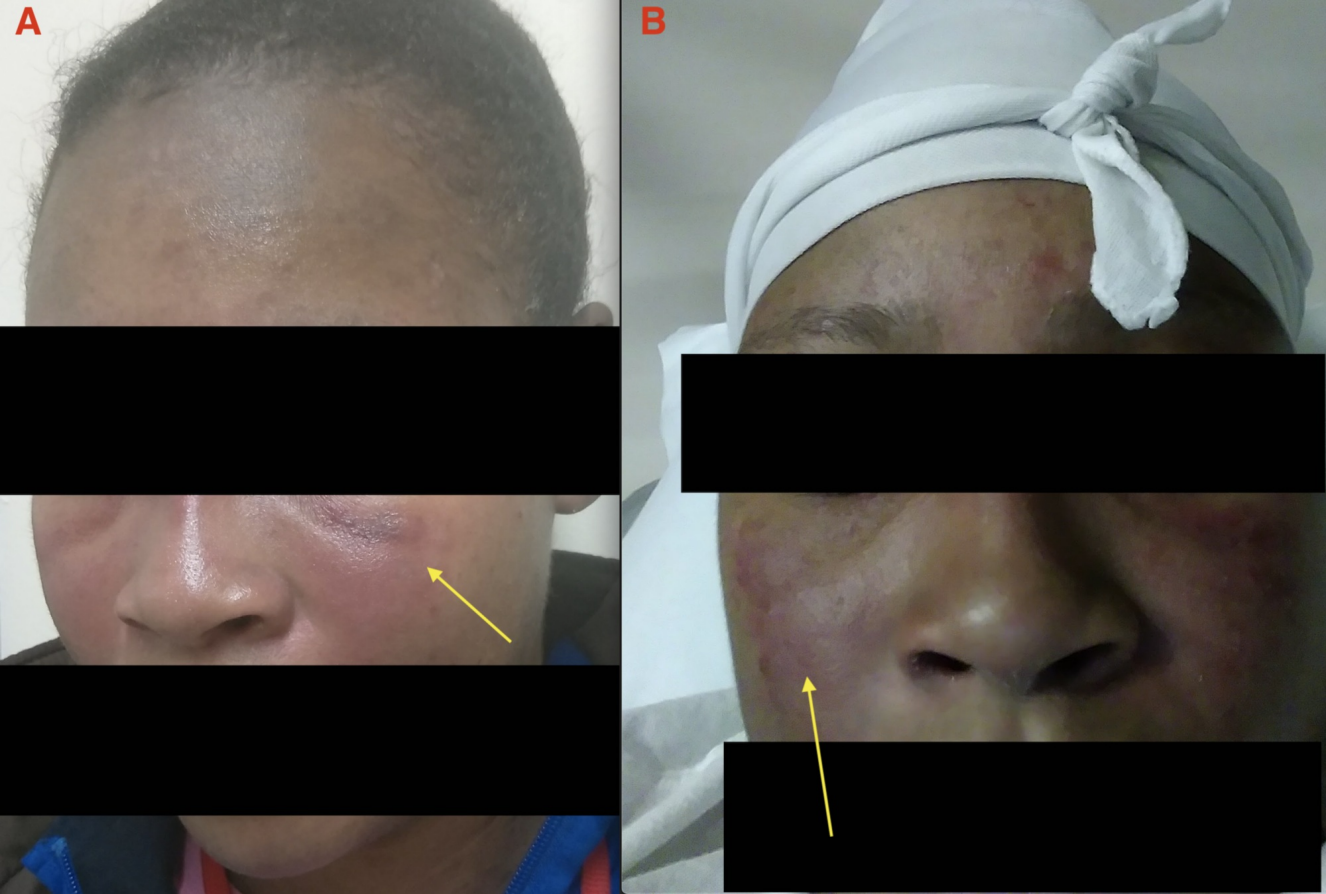
Hypopigmented and erythematous lesions on the face and cheeks (yellow arrows).

**Figure 2:**
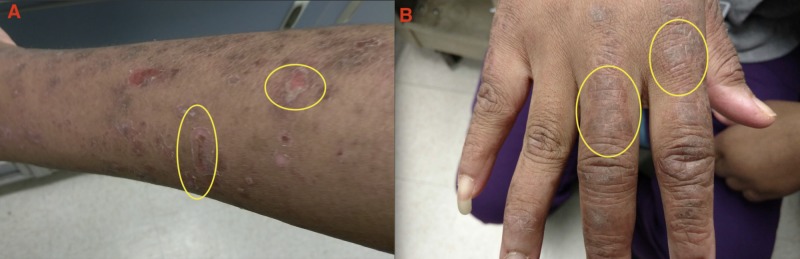
Hyperpigmented macules on the extremities (A) and papules with stable thick scales on the dorsum of the hand (B) (yellow circles).

The patient presented to our care in August 2015 with a chief complaint of persistent productive cough and shortness of breath. The patient reported the following associated symptoms: fever, malaise, swollen glands, headache, dysphagia, ear, and sinus pain. Physical examination was remarkable for scaly hypopigmented and erythematous lesions over the neck and nasal region (Figure 1). In addition, hyperpigmented macules were present on her arms and legs with overlying scales, and papules with a stable thick scale on the dorsum of hands (Figure 2). The patient underwent extensive laboratory workup in August 2015, which reported normal levels of anti-double-stranded DNA, anti-Smith, anti-nuclear ribonucleoprotein, anti-Sjögren's-syndrome-related antigens A and B, anti-topoisomerase I (anti-Scl 70), anti-histidyl transfer RNA synthetase (anti-Jo-1), anti-myeloperoxidase, and anti-proteinase 3. 

**Figure 3:**
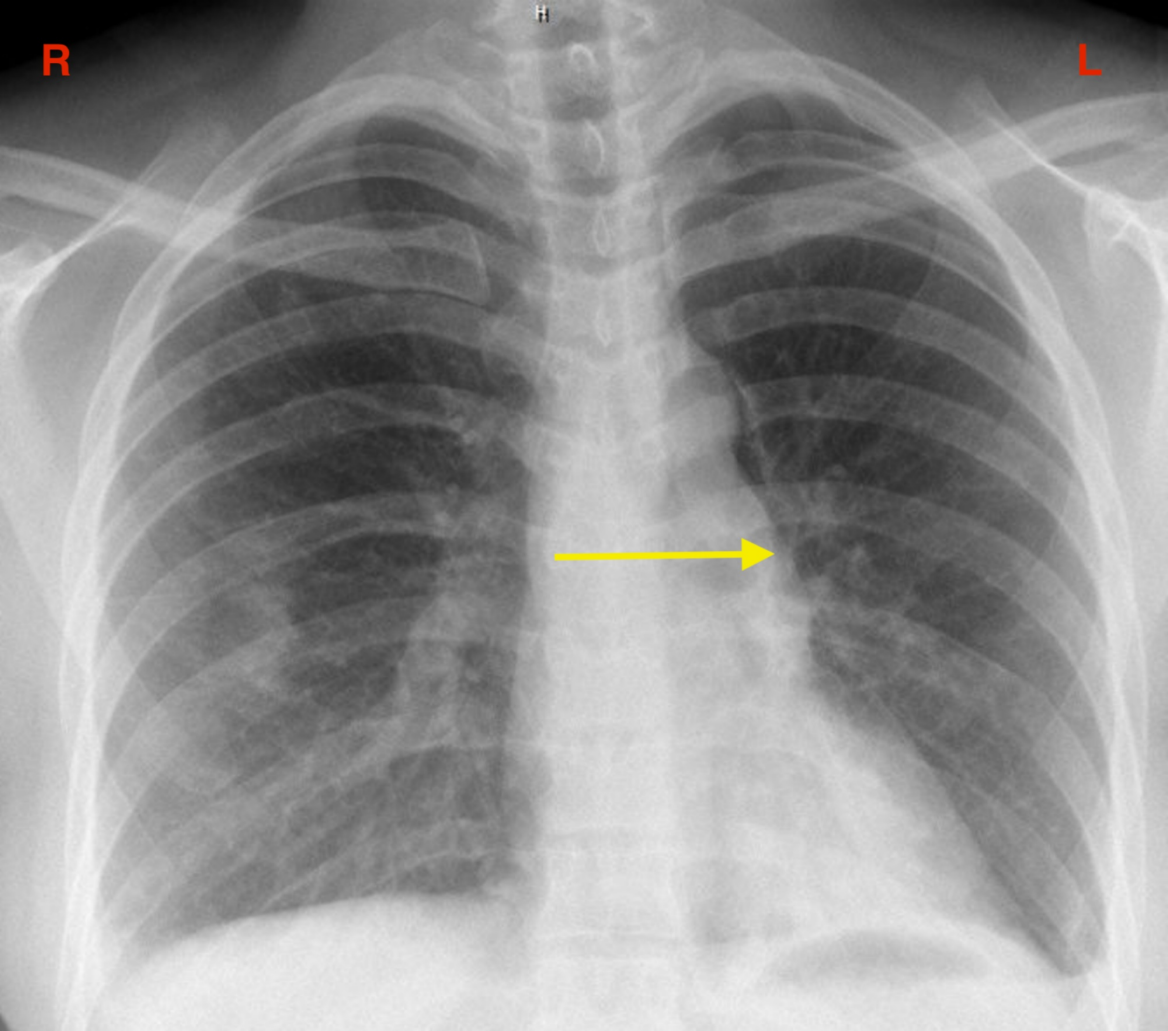
Opacification in the left lung (yellow arrow).

Chest x-ray depicted persistent opacification in the left lung despite receiving amoxicillin-clavulanate, azithromycin, and prednisone (Figure 3). She then underwent a chest computed tomography (CT) scan without contrast, which illustrated bilateral patchy alveolar consolidation prominent at the left lower lobe. Due to persistent symptoms, she underwent bronchoscopy with multiple transbronchial biopsies and brushings of the left lower lobe. The collected specimen was sent for analysis, and pathology showed microfocus organizing pneumonitis with a focus of reactive pneumocytes along with sclerosis and vacuolated macrophages. Also, Gomori methenamine-silver nitrate stain and acid-fast stain (also known as the Ziehl-Neelsen stain) were negative for Pneumocystis jiroveci and Mycobacterium species, respectively. Specimen analysis, along with negative staining, suggested organizing pneumonia as the culprit contributing to her symptoms. The patient was started on prednisone 40 milligrams (mg) and sulfamethoxazole-trimethoprim for pneumocystis pneumonia prophylaxis, which provided moderate relief from the symptoms. Furthermore, the patient was prescribed azathioprine. The patient was frequently reevaluated for her signs and symptoms. The patient discontinued prednisone in January 2016 after taking it for three months. Over the next few months, the patient had frequent relapses, prompting the restart of prednisone at a lower dose. 

**Figure 4:**
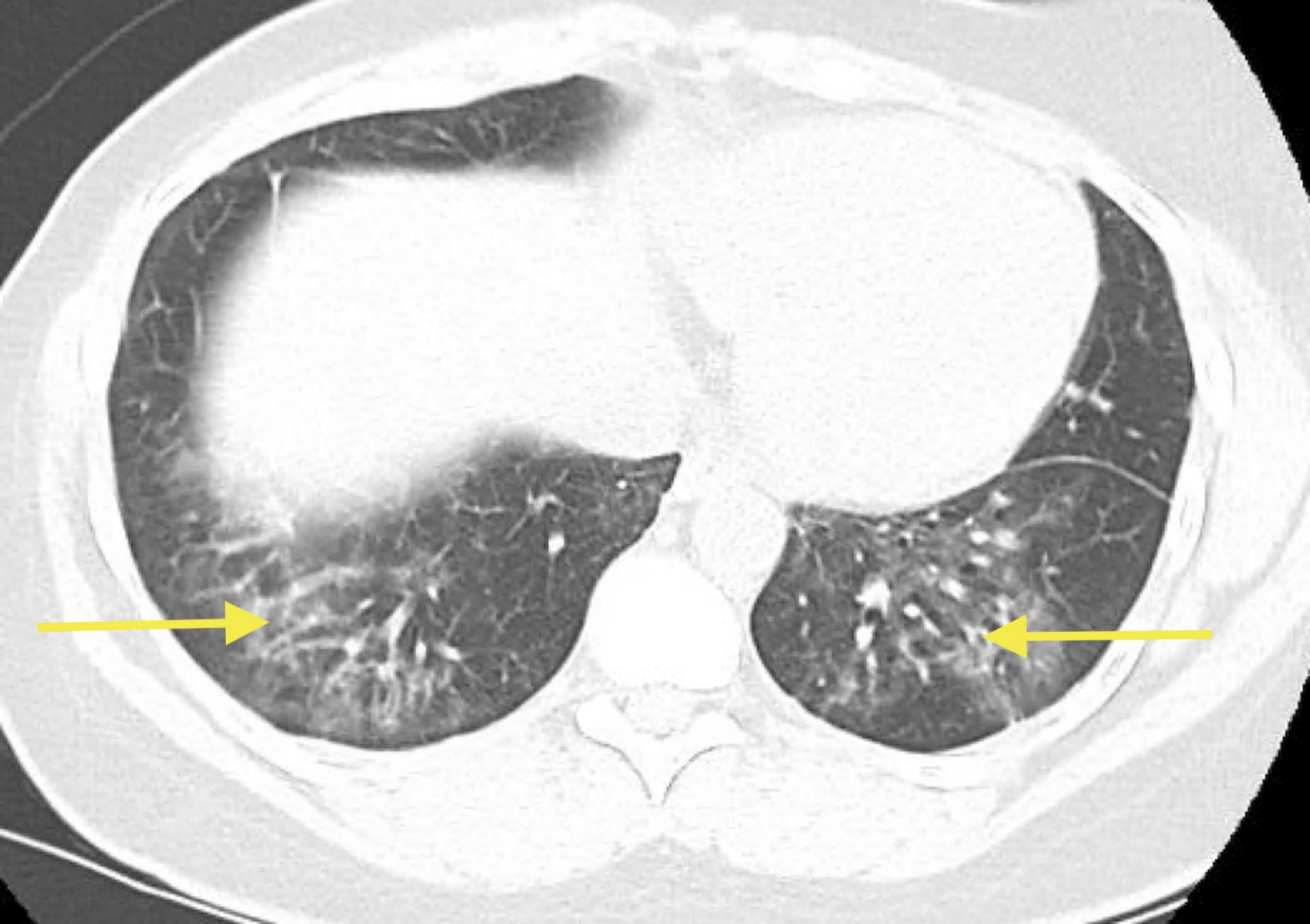
Bilateral mid-lower lung zone reticulonodular opacities (yellow arrows).

In early 2016, the patient reported some clinical improvement along with stabilization of shortness of breath and cough. A chest CT was ordered, which showed improvement of bilateral consolidation in comparison to the previous chest CT. The patient was eventually referred to a rheumatologist. Another chest CT ordered in August 2016 showed stable interstitial changes and improvement of bilateral patchy airspace consolidation, in contrast to previous imaging. In addition to radiographic improvement, the patient denied feeling shortness of breath with normal ambulation on a flat surface. However, she started to have difficulty climbing stairs, which was ultimately contributed to arthralgia and not to dyspnea. Despite overall improvement, interstitial changes remained more prominent in the left lower lung field versus the right lower lung field. Although the patient experienced overall clinical improvement, her symptoms, particularly shortness of breath, returned in early 2017 as an attempt was made to decrease her prednisone dose. This was done to control her avascular necrosis of the hip, which was contributed to chronic prednisone use. At that time, the patient was referred to an ILD specialist for further expertise and opinion. She subsequently underwent esophagogastroduodenoscopy (EGD), revealing reflux esophagitis and gastritis without hemorrhage and duodenitis. In May 2017, skin biopsy was remarkable for vacuolar interface changes with hyperkeratosis, dyskeratosis, pigment incontinence, and post-inflammatory hyperpigmentation without psoriasis. Early CT images were reviewed, and assessments suggesting bilateral mid-lower lung zone reticulonodular opacities with ground glass opacities were made, on par with previously described findings (Figure 4). ILD specialists echoed and confirmed the findings and diagnosis of dermatomyositis with ILD. EGD mentioned earlier, and colonoscopy was performed to rule out potential malignancy due to the known association between dermatomyositis with malignancies of the lung, ovary, gastrointestinal, and lymphoma. 

Currently, the patient is being managed by a team of multidisciplinary physicians, including physical therapy. The comprehensive management approach has stabilized patients' symptoms and allowed her to continue with daily activities. The patient has been prescribed prednisone 10 mg, mycophenolate mofetil 1,500 mg, duloxetine 60 mg, fludrocortisone 0.1 mg, levothyroxine 0.15 mg, nortriptyline 75 mg, oxycodone, and acetaminophen 7.5-325 mg, along with intravenous immunoglobulin.

## Discussion

COP is a rare ILD that can be idiopathic or secondary to a preexisting chronic inflammatory condition, such as dermatomyositis. Here, we presented a patient with a history of dermatomyositis who suddenly developed shortness of breath. Although rare, cases of COP occurring with dermatomyositis have been published [1,2]. Clinical evidence suggests that most patients with ILD present with severe shortness of breath and non-productive cough, which can manifest itself quickly, like our patient [3,4].

Although the precise incidence and prevalence data of this condition are not readily available, studies suggest meaning annual incidence to be approximately 1.1 per 100,000 [5,6]. The pathogenic mechanism involves alveolar injury leading to the spilling of plasma proteins into the alveolar lumen triggering the recruitment of fibroblasts along with deposition of fibrin. Excessive proliferation of fibrous tissue along with vascular endothelial growth factor and matrix metalloproteinases has been noted to be the central figure in this condition [7,8].

Patients with this condition typically present in the fifth or sixth decades of life, with equally affecting men and women [5,9]. Patients usually present with flu-like symptoms such as fever, malaise, cough, and shortness of breath. Our patient was asymptomatic until April 2015; her symptoms had an acute onset resembling typical flu. Furthermore, clinical evidence suggests that the majority of the patients experience symptoms for less than two months [10-12]. However, in some patients, symptoms can persist.

Although there are no specific laboratory tests or imaging to diagnose this condition, the patient should be evaluated with routine workup, including complete blood count, liver and kidney studies, and inflammatory markers such as erythrocyte sedimentation rate [13,14]. Also, the literature recommends diagnostic studies to rule out viruses and other conditions, along with testing for various connective tissue diseases [15].

The patient presented to our care in August 2015 with acute onset of shortness of breath and productive cough. After ruling out common causes of shortness of breath and productive cough, the patient was worked up for chronic inflammatory conditions using routine laboratory and radiographic studies to work through a list of differential diagnoses. Initially, the patient had a pulmonary function test exhibiting a moderate restrictive pattern, which trended towards normal range as management progressed. The last office spirometry revealed improvement in her forced expiratory volume in one second and forced vital capacity of 76% and 73%, respectively (Table 1). 

**Table 1:** Pulmonary function test results over the years. FEV-1: forced expiratory volume in one second; FVC: forced vital capacity.

Date	FEV-1 (%)	FVC (%)	FEV-1/FVC (%)
08/26/2019	76	73	104
01/28/2019	73	72	101
07/26/2018	76	69	111
10/12/2017	71	66	107
01/05/2017	70	69	101
05/03/2016	79	77	102
09/23/2015	65	66	98

.

## Conclusions

COP is a rare condition that can be commonly mistaken for other forms of ILD. Presenting symptoms are often seen with other acute illnesses and further confuse the initial presentation. A persistent or progressive clinical picture often leads to a more extensive workup and eventual diagnosis of COP, a diagnosis of exclusion. If an underlying inflammatory or connective tissue disease is connected to this pathology, treatment with steroids, immunosuppressants, or biologics is required to stabilize the condition. The timely diagnosis and treatment of our patient have provided much-needed relief along with allowing her to resume daily activities without significant restrictions. 
